# *Burkholderia phytofirmans* PsJN induces long-term metabolic and transcriptional changes involved in *Arabidopsis thaliana* salt tolerance

**DOI:** 10.3389/fpls.2015.00466

**Published:** 2015-06-23

**Authors:** Ignacio Pinedo, Thomas Ledger, Macarena Greve, María J. Poupin

**Affiliations:** ^1^Laboratorio de Bioingeniería, Facultad de Ingeniería y Ciencias, Universidad Adolfo IbáñezSantiago, Chile; ^2^Center for Applied Ecology and SustainabilitySantiago, Chile

**Keywords:** plant growth promoting rhizobacteria (PGPR), ion transport, osmotic stress response, priming, abiotic stress tolerance, reactive oxygen species (ROS), rhizosphere, beneficial bacteria

## Abstract

Salinity is one of the major limitations for food production worldwide. Improvement of plant salt-stress tolerance using plant-growth promoting rhizobacteria (PGPR) has arisen as a promising strategy to help overcome this limitation. However, the molecular and biochemical mechanisms controlling PGPR/plant interactions under salt-stress remain unclear. The main objective of this study was to obtain new insights into the mechanisms underlying salt-stress tolerance enhancement in the salt-sensitive *Arabidopsis thaliana* Col-0 plants, when inoculated with the well-known PGPR strain *Burkholderia phytofirmans* PsJN. To tackle this, different life history traits, together with the spatiotemporal accumulation patterns for key metabolites and salt-stress related transcripts, were analyzed in inoculated plants under short and long-term salt-stress. Inoculated plants displayed faster recovery and increased tolerance after sustained salt-stress. PsJN treatment accelerated the accumulation of proline and transcription of genes related to abscisic acid signaling (*Relative to Dessication*, *RD29A* and *RD29B*), ROS scavenging (*Ascorbate Peroxidase 2*), and detoxification (*Glyoxalase I 7*), and down-regulated the expression of *Lipoxygenase 2* (related to jasmonic acid biosynthesis). Among the general transcriptional effects of this bacterium, the expression pattern of important ion-homeostasis related genes was altered after short and long-term stress (*Arabidopsis K^+^ Transporter 1*, *High-Affinity K^+^ Transporter 1*, *Sodium Hydrogen Exchanger 2*, and *Arabidopsis Salt Overly Sensitive 1*). In all, the faster and stronger molecular changes induced by the inoculation suggest a PsJN-priming effect, which may explain the observed tolerance after short-term and sustained salt-stress in plants. This study provides novel information about possible mechanisms involved in salt-stress tolerance induced by PGPR in plants, showing that certain changes are maintained over time. This opens up new venues to study these relevant biological associations, as well as new approaches to a better understanding of the spatiotemporal mechanisms involved in stress tolerance in plants.

## Introduction

Salinity is one of the most limiting factors in agriculture, affecting more than 45 million ha of irrigated land worldwide (Munns and Tester, 2008). It reduces plant growth and crop quality, with estimated annual global costs equivalent to US$ 150 million in 2011 (FAO, 2011). Hence, increased salt tolerance of crops and horticultural species is needed to sustain the growing demand for food production in many regions of the world (Gupta and Huang, 2014). In the early stages of stress, salinity affects osmotic potential in plants, limiting their water uptake (Munns, 2005). If salt exposure is prolonged, ions (mainly Na^+^) accumulate inside the cells causing toxicity (Munns, 2005; Munns et al., 2006) and growth decrease by impairing metabolic processes and reducing photosynthetic efficiency (Deinlein et al., 2014). As a result of both osmotic and ionic stress, plants accumulate reactive oxygen species (ROS) and toxic compounds that may lead to cell death (Mittler, 2002; Ismail et al., 2014).

Plants have evolved various mechanisms to cope with this stress (Munns and Tester, 2008; Zhang and Shi, 2013; Gupta and Huang, 2014) and it has been recently proposed that the timing of their response to salt-stress is one of the key factors that influences the ultimate response outcome, ranging from adaptation to plant death (Geng et al., 2013; Ismail et al., 2014). At the onset of stress, there is an early response characterized by a growth arrest that involves detoxification activity, ROS scavenging, a general hormonal response mainly related with abscisic acid (ABA) and the adjustment of osmotic potential (Ismail et al., 2014). ROS scavenging is carried out by enzymes such as ascorbate peroxidases (Davletova et al., 2005; Abogadallah, 2010; Bharti et al., 2013), superoxide dismutase, catalase, glutathion reductase, and glutathion *s*-transferase (Apel and Hirt, 2004; Gill and Tuteja, 2010). On the other hand detoxification is related to crucial pathways, such as the glyoxalase pathway that detoxifies methylglyoxal (Mustafiz et al., 2011; Kwon et al., 2013). ABA response is characterized by the expression of various genes such as *RD29A* and *RD29B* related to several abiotic stresses (Yamaguchi-Shinozaki and Shinozaki, 1994; Hu et al., 2013). Finally, osmotic potential adjustment is regulated by the accumulation of proline and other compatible solutes (Sneha et al., 2013).

The second phase of the response to salinity is caused by the accumulation of salt ions at toxic levels and needs more time to develop (Munns, 2005; Shavrukov, 2013; Ismail et al., 2014). To protect themselves against Na^+^ toxicity, plants have specific tissue-dependent mechanisms that minimize ion entry into cells, as well as Na^+^ exclusion or storage into vacuoles (Lv et al., 2012; Fan et al., 2014) and/or in older leaves (Munns, 2002; Munns and Tester, 2008). This is achieved by the activity of diverse ion transporters, in a complex mechanism that is not completely understood (Rozema and Schat, 2013; Zhang and Shi, 2013; Deinlein et al., 2014; Gupta and Huang, 2014). It has been proposed that HKT1, a plasma membrane Na^+^ transporter, localized mainly in the xylem parenchyma cells (XPC), acts by unloading Na^+^ from the xylem sap into XPC in the roots, preventing excessive amounts of Na^+^ from reaching the shoots (Munns et al., 2012). It is believed that the excess of sodium in the XPC is excluded to the apoplast by the plasma membrane Na^+^/H^+^ antiporter SOS1 (Shi et al., 2002; Ariga et al., 2013). The vacuolar storage of sodium in almost all cells is conducted mainly by vacuolar Na^+^/H^+^ antiporters of the NHX family (Kronzucker and Britto, 2011). Recently, a possible alternative mechanism for sodium compartmentalization via vesicle trafficking has been also proposed (Garcia de la Garma et al., 2015).

Many studies have shown the use of plant growth promoting rhizobacteria (PGPR, Kloepper and Schroth, 1981) as a valuable strategy to improve plant growth (Reviewed in Lugtenberg and Kamilova, 2009) and, more recently, to confer abiotic stress tolerance in plants (Egamberdieva et al., 2008; Zahir et al., 2008; Kaymak et al., 2009; Yang et al., 2009; Upadhyay et al., 2012; Chang et al., 2014; Naveed et al., 2014). In relation to salt-stress, it has been shown that the inoculation of PGPR in some plant species promotes growth and nutrient uptake under saline conditions (Mayak et al., 2004; Dodd and Perez-Alfocea, 2012; Han et al., 2014). In maize (*Zea mays*), inoculation with *Pseudomonas* sp. promotes growth and increases the chlorophyll content of plants exposed to salinity (Nadeem et al., 2007). In cotton (*Gossypium hirsutum*), treatment with *Klebsiella oxytoca* promotes growth and nutrient uptake of plants grown in saline media (Liu et al., 2013). The identification and exploitation of these microorganisms that interact with plants by alleviating stress opens new alternatives for developing a strategy against salinity challenge, as well as novel approaches to discover hitherto unknown pathways involved in stress tolerance (Dodd and Perez-Alfocea, 2012). Nevertheless, the molecular mechanisms underlying these plant–bacteria interactions under salt-stress are far from being understood. To tackle this issue, the salt-sensitive *Arabidopsis thaliana* ecotype Col-0 plants and the well-known PGPR *Burkholderia phytofirmans* PsJN, were used as model organisms in this study. The phenotypical effects of PsJN strain inoculation were studied under short and long-term salt-stress in plants, considering their whole life cycle, finding that inoculation increased plant growth and tolerance to various salt concentrations. Also, spatiotemporal molecular and biochemical responses of *A. thaliana* plants exposed to salt-stress were investigated in the early and later stages of stress. Amongst the changes produced by bacterial treatment, a promotion of metabolic and transcriptional responses associated to salt tolerance was noted. These changes included a faster accumulation of proline, accelerated induction of general abiotic stress responsive genes, and a transcriptional regulation of genes involved in ion homeostasis during salt-stress. Interestingly, some of these responses were maintained over time after long-term exposure to salinity. The anticipation of the molecular response to salt-stress along with the transcriptional changes in ion transporter genes could explain, at least in part, the improved salt stress tolerance of inoculated plants along their whole life cycle. To the best of our knowledge, this is one of the first reports of a detailed molecular and phenotypical analysis of the spatiotemporal responses of plants under salt-stress inoculated with a PGPR.

## Materials and Methods

### Plant Growth Conditions and Treatments

*Burkholderia phytofirmans* PsJN, kindly provided by Angela Sessitsch (AIT, Austria), was routinely grown in liquid minimal saline medium containing 10 mM fructose, in an orbital shaker (150 rpm) at 30°C. Cell suspensions from each inoculum were then collected and adjusted to approximately 10^8^ colony forming units per milliliter (CFU/ml), as determined by plate counting. Col-0 *A. thaliana* seeds were obtained from the ABRC. Seeds were surface sterilized with 50% sodium hypochlorite (100% commercial laundry bleach containing 0.1% Tween 20, rinsed three times with sterile water, and kept at 4°C for 7 days to synchronize germination. Square Petri dishes were prepared with half strength Murashige and Skoog (1962) medium (MS1/2) 0.8% agar. To prepare the inoculated plates, the initial inoculum (10^8^ CFU/ml) was homogenously diluted in MS1/2 0.8% agar just before gelling to reach a final concentration of 10^4^ CFU per ml of medium. Then, sterilized and synchronized seeds were sown in the Petri dishes with MS1/2 medium inoculated or not with the strain. To assess the effect of inactivated bacteria, an inoculum was heated at 95°C for 20 min and then used at the same dilution of 10^4^ CFU per ml of MS1/2 medium (Poupin et al., 2013). Mortality was corroborated by plate counting. Plates were placed vertically in a growth chamber at 22°C with a photoperiod of 16/8 h (light/dark). At day 11 after sowing (11 DAS) plants were transplanted to MS1/2 with saline concentration ranging from 0 mM NaCl/0 mM CaCl_2_ to 250 mM NaCl/25 mM CaCl_2_. After 7 days in saline media different growth parameters were determined in plants. For the recovery treatment plants inoculated and stressed as described before were transferred to individual pots with a 2:1 mix of peat/vermiculite at 18 DAS and were watered normally. For the long-term salt stress seeds were sown on MS1/2 (0.8% agar) inoculated or not as described above and at 11 DAS were transferred to individual pots with a 2:1 mix of peat/vermiculite. After 7 days of acclimation, plants were irrigated two times per week with 150 mM NaCl/15 mM CaCl_2_ and one time with water.

### Plant Growth Measurements and Statistical Analysis

Fresh weight of plants was determined with a Shimadzu analytical balance (Shimadzu Corporation, Japan). The plants in soil were photographed every 2 days, starting 7 days after transplantation; rosette area color patterns were calculated using Adobe Photoshop Cs3 software (Adobe Systems Incorporated, San Jose, CA, USA). Senescent leaves were considered as those with at least 1/3 of their area with senescence signs. Stem length was registered using a ruler. T-student was used to compare rosette area between stressed inoculated and non-inoculated plants. When the experiments considered two factors (bacteria and salt) two-way ANOVA was used. Kolmogorov–Smirnov test was used for normality evaluation, and Hartley and Bartlett test for homogeneity of variances evaluation. Statistical analyses were carried out using the General Linear Models option in the statistical software Prism Graphpad 5 (GraphPad Software, Inc., La Jolla, CA, USA). When differences in the means were significant, a Bonferroni correction test was performed. Bonferroni correction was applied to determine which treatments were significantly different from others.

### Proline Extraction and Measurement

Proline extraction was performed according to a modification of Bates et al. (1973) method. Plantlets were collected 2, 24, and 48 h after salt-stress. For each treatment seven to ten plants were used per replicate, and three to six replicates were used per treatment. Plants were weighed on an analytic balance (Shimadzu Corporation, Japan) and grounded with a pestle in an eppendorf tube containing a sulfosalicylic acid 140 mM solution. The resulting solution was filtered through a N°1 filter paper (11 μm; GE Healthcare, Hartford, CT, USA). This liquid extract was then mixed with an acid ninhydrin 140 mM solution and glacial acetic acid in a 1:1:1 proportion. Tubes were heated at 100°C for one hour and cooled on ice. Toluene was added in a 1:1 proportion, and the reaction mixture was vigorously agitated. Organic fraction was separated and added to a quartz cuvette for absorbance measurement of the ninhydrin-proline complex at 520 nm by spectrophotometry. A proline calibration curve was performed using 10, 20, 25, 40, 50, 75, 100, 150, and 200 μg of commercial proline. Finally, values were corrected using the following formula:

$$\mathrm{Pr}oline\,\;\left(\mu g{\;g}^{-1}\right)=\frac{A_{520}}{0.0489\times g}$$

### RNA Extraction, cDNA Synthesis, and qRT-PCR Analyses

For short-term stress experiments, plants were treated as described. RNA extractions were performed on plantlets before being transplant to saline media (150 mM NaCl/15 mM CaCl_2_), and 2, 24, or 72 h after transplant. About 50 plantlets per treatment were separated in five groups; roots; and rosettes were separated and collected in different eppendorf tubes. For long-term stress experiments plants were transferred to soil as described before, and RNA extraction was performed at 46 DAS. Five plants were used per treatment, and in each one the oldest and the newest leaves were collected in different eppendorf tubes. Then, RNA was obtained using the Trizol^®^ (Invitrogen^TM^, USA) method following the manufacturer’s instructions. For cDNA synthesis, 1 μg of total RNA treated with DNAse I (RQ1, Promega, USA) was reverse transcribed with random hexameric primers using the Improm II reverse transcriptase (Promega, USA), according to the manufacturer’s instructions. Real time (RT)-PCR was performed using the Brilliant^®^ SYBR^®^ Green QPCR Master Reagent Kit (Agilent Technologies, USA) and the Eco RT PCR detection system (Illumina^®^, USA) as described by Poupin et al. (2013). The PCR mixture (10 μl) contained 2.0 μl of template cDNA (diluted 1:10) and 140 nM of each primer. Amplification was performed under the following conditions: 95°C for 10 min, followed by 40 cycles of 94°C, 30 s; 58–60°C, 30 s; and 72°C, 30 s, followed by a melting cycle from 55 to 95°C. Relative gene expression calculations were conducted as described in the software manufacturer’s instructions: an accurate ratio between the expression of the gene of interest (GOI) and the housekeeping (HK) gene was calculated according to equation: 2^-(ΔCtGOI-HK)^ (Dauelsberg et al., 2011). Then, gene expression levels were normalized to the average value of the treatment with less expression. Expression of three HK genes was analyzed for treatments *AtSAND* (At2g28390), *PP2A* (At1g13320), and *TIP41-like* (At4g34270), using previously described PCR primers In all cases, expression of HK genes was highly stable and similar results were obtained using them as normalization genes (Czechowski et al., 2005). Data presented here represent normalization using *AtSAND* amplification. Primers were designed using Primer Express v.2.0 (Applied Biosystems, USA) and confirmed with Primer-BLAST (NCBI). Sequences of all primers and their references (if applicable) are listed in Supplementary **Table S1**. In all cases the reaction specificities were tested with melt gradient dissociation curves and electrophoresis gels (agarose 2%) of each PCR product. All experiments were performed with three to five biological and two technical replicates.

## Results

### *Burkholderia phytofirmans* PsJN Enhances Salt-Stress Tolerance and Recovery of Stressed *A. thaliana* Plants

**FIGURE 1:**
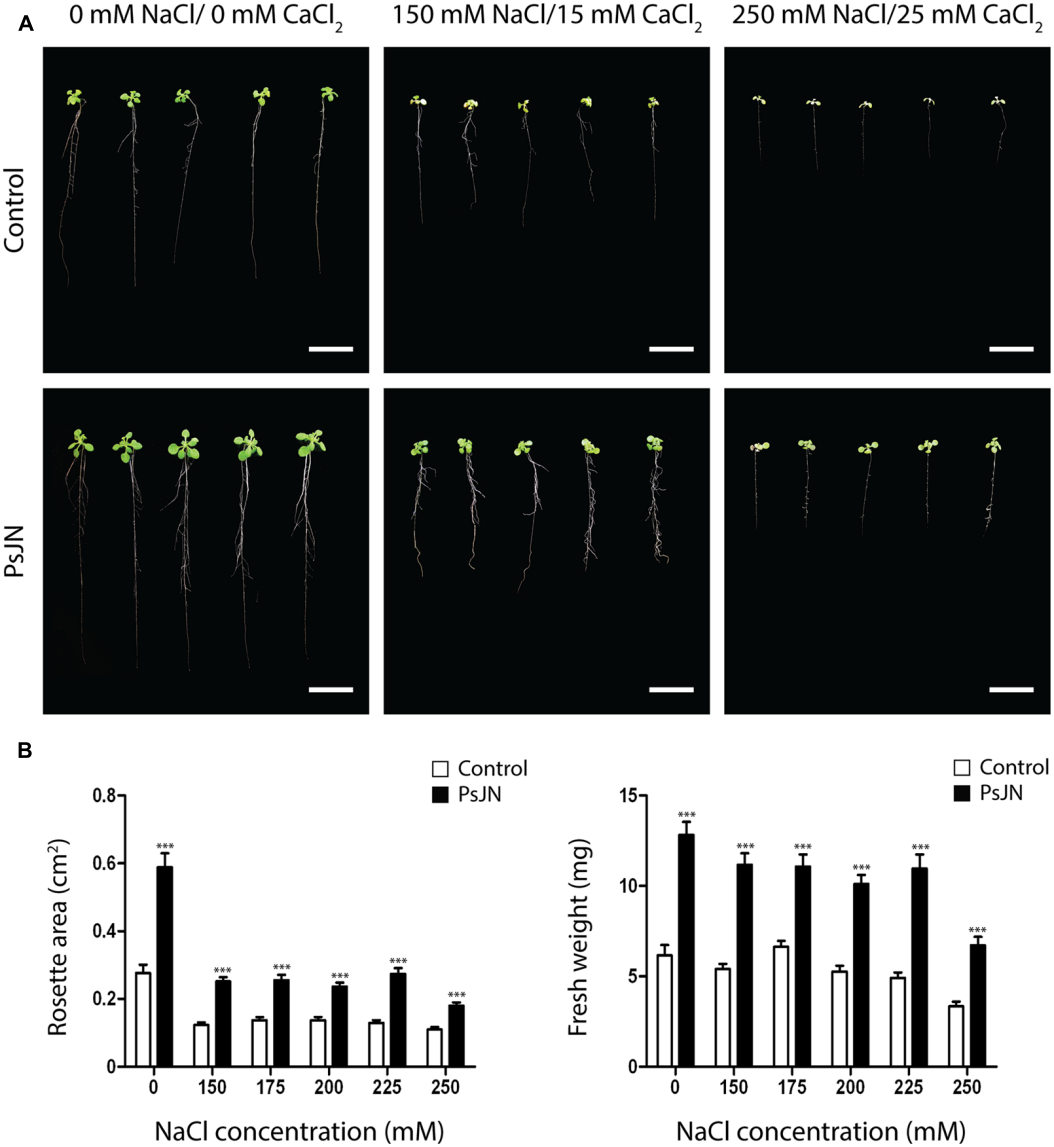
**Effects of *Burkholderia phytofirmans* PsJN on *Arabidopsis thaliana* growth *in vitro*. (A)** Representative photographs of *A. thaliana* plants treated with or without *B. phytofirmans* PsJN, and transplanted at 11 days after sowing (DAS) to Murashige Skoog (MS) with or without addition of salt (150 mM NaCl/15 mM CaCl_2_ to 250 mM NaCl/25 mM CaCl_2_). Data was collected 7 days after transplantation. **(B)** Graphic representation of rosette area (left) and fresh weight (right) of plants treated under the experimental conditions described before. Data are means ± 1 SE of at least 20 plants per treatment. Asterisks indicate significant differences between control and PsJN treatment (Two-way ANOVA, *p* < 0.05; Bonferroni test, ^∗∗∗^*P* < 0.01). Results are representative of two different experiments. White bars in photographs correspond to 2 cm.

To address for differences in growth of *A. thaliana* Col-0 plants exposed to salt-stress *in vitro*, seeds were sown in half strength Murashige and Skoog (1962) media (MS1/2) with or without inoculation of strain PsJN as described in the “Materials and Methods” section. At 11 DAS, plants were transferred to MS1/2 media containing different salt concentrations ranging from 150 mM NaCl/15 mM CaCl_2_ to 250 mM NaCl/25 mM CaCl_2_. Seven days after the transplant plants were photographed (**Figure 1A**) and rosette areas and fresh weights were determined (**Figure 1**). *B. phytofirmans* treatment produced a significant 87 ± 20% increase in rosette area in stressed plants (**Figure 1B**, left). Fresh weight was also significantly higher (97 ± 21%) in plants treated with strain PsJN and exposed to salinity (**Figure 1B**, right). Also, primary root length was increased in inoculated and stressed plants (Supplementary **Figure S1**). In addition, a treatment with heat-killed bacteria (K-PsJN) was incorporated as described by Poupin et al. (2013), to discriminate the effects of metabolically active bacteria from those of inactive bacteria on plants under salt-stress. Treatment with K-PsJN had no effect on *A. thaliana* growth, neither on MS media nor under salt-stress (Supplementary **Figure S1**).

**FIGURE 2:**
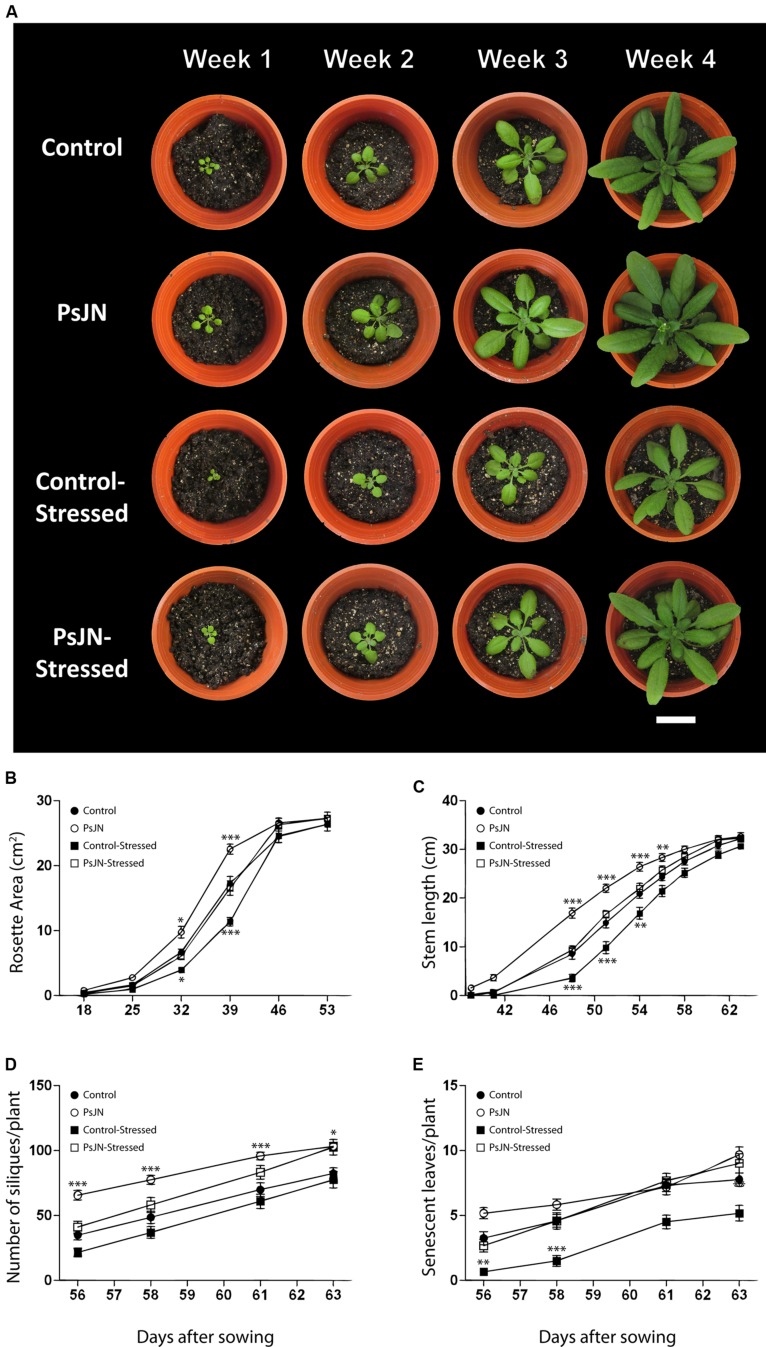
**Effect of *B. phytofirmans* PsJN on *A. thaliana* recovery after salt stress. (A)** Representative photographs of *in vitro* salt-treated *A. thaliana* plants transplanted to soil. Plants were sown in half strength MS media with or without inoculation of *B. phytofirmans* PsJN. Eleven DAS plantlets were transplanted to MS media with or without additional 150 mM NaCl/15 mM CaCl_2_. Seven days after saline treatment, plants where transplanted to soil. **(B–E)** Graphic representation of average rosette area **(B)**, average stem length **(C)**, average number of siliques/plant **(D)**, and average number of senescent leaves/plant **(E)** of plants under the experimental conditions described before. Data are means ± 1 SE of at least 12 plants per treatment. Asterisks indicate significant differences between control treatment and the other treatments in each time (Two-way ANOVA, *p* < 0.05; Bonferroni test, ^∗^*P* < 0.1; ^∗∗^*P* < 0.05; ^∗∗∗^*P* < 0.01). White bar in photograph corresponds to 2 cm.

To investigate the effects of PsJN inoculation on the recovery of *A. thaliana* plants exposed to salt-stress, plants were inoculated and exposed to salinity as described in the “Materials and Methods” section. After 7 days in the saline media, plants were transferred to soil and watered normally. Plant growth was recorded during 2 months (**Figure 2A**) by the measurement of rosette area, stem length, number of siliques, and senescent leaves (**Figures 2B–E**, respectively). During the first month in soil, plants treated with strain PsJN and not stressed had significantly larger rosette area, compared to the other treatments. Plants inoculated with strain PsJN and exposed to stress showed no differences in comparison to the control plants (non-inoculated and non-exposed to salt), while the non-inoculated and stressed plants had significantly smaller rosettes than all the other treatments. This pattern was observed until 46 DAS, when plants in all treatments showed comparable rosette areas (**Figure 2B**).

Stems appeared first (∼38 DAS) in plants formerly treated with strain PsJN and not exposed to salt-stress, followed by both, control and inoculated salt-stressed plants (∼40 DAS), and finally by the non-inoculated and stressed plants (∼42 DAS; **Figure 2C**). Non-stressed plants treated with strain PsJN always showed significantly longer stems than plants from the other treatments until 58 DAS. At this point, control and inoculated salt-stressed plants reached the same stem length as PsJN-treated non-stressed plants. Finally, the non-inoculated salt-stressed plants reached the same stem length as the other treatments at 61 DAS (**Figure 2C**).

At 56 DAS, all the plants corresponding to the four treatments developed at least one silique. From then on, non–stressed plants previously treated with strain PsJN always showed a significantly higher number of siliques than the other treatments. At 63 DAS, both stressed and non-stressed plants, previously treated with strain PsJN, reached a comparable number of siliques, which was significantly higher (25%) than the control plant’s number of siliques. Non-inoculated plants, stressed, and non-stressed, showed a comparable number of siliques through the treatment (**Figure 2D**). At 61 DAS, all the treatments exhibited at least one senescent leaf per plant. Plants stressed and non-treated with strain PsJN, presented significantly less senescent leaves than all the other treatments at every measured time (**Figure 2E**). This behavior was maintained throughout the whole experiment (**Figure 2E**).

**FIGURE 3:**
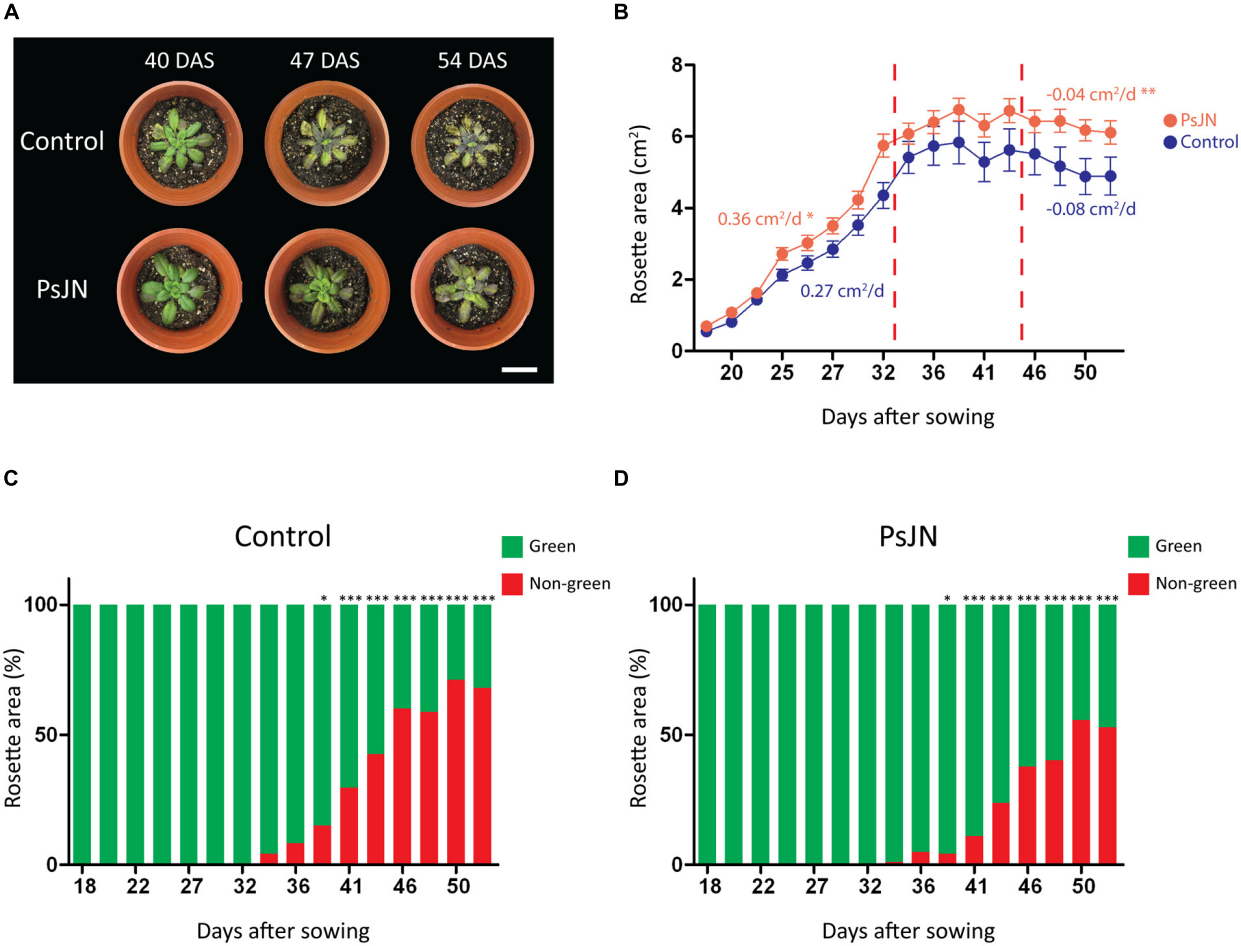
**Effects of *B. phytofirmans* PsJN on *A. thaliana* long-term growth in saline media. (A)** Representative photographs of *A. thaliana* plants treated with or without *B. phytofirmans* PsJN, and transplanted at 11 DAS to individual pots with a 2:1 mix of peat/vermiculite and irrigated periodically with saline solution containing 150 mM NaCl/15 mM CaCl_2_. **(B)** Graphic representation of average rosette area (growth rate is indicated for the first and last periods of the experiment, cm^2^/d. **(C,D)** Percentage of non-green area of non-inoculated **(C)** and inoculated **(D)** plant rosettes under the experimental condition described. Data are means ± 1 SE of at least eight plants per treatment. Asterisks indicate significant differences between control and PsJN treatment in each time point (*t*-student, *p* < 0.05; Welch’s correction, ^∗^*P* < 0.1; ^∗∗^*P* < 0.05; ^∗∗∗^*P* < 0.01). Whitebars in photograph correspond to 2 cm.

To explore a long-term salt-stress tolerance in *Arabidopsis*, plants were inoculated as described in the “Materials and Methods” section, transferred to soil and stressed by periodically irrigating with a saline solution (150 mM NaCl/15 mM CaCl_2_). Rosette areas were higher in inoculated plants after 35 days of stress (**Figures 3A,B**). During the first 14 days under stress, growth rate of inoculated plants was significantly higher than the non-inoculated plants (0.36 cm^2^/d vs. 0.27 cm^2^/d, respectively; **Figure 3B**). During the final stage of the stress treatment the rosette area of non-inoculated plants decreased (wilted) in a significantly higher rate (-0.08 cm^2^/d) than the inoculated plants (-0.04 cm^2^/d; **Figure 3B**). Also, stressed foliar areas were determined analyzing the color pattern of plant images, to quantify the recession of green colored leaf area caused by stress. Interestingly, inoculated plants showed significantly larger green areas than the non-inoculated plants (**Figures 3C,D**). Control non-stressed plants began to lose green coloration only at 50 DAS (data not shown).

### *Burkholderia phytofirmans* PsJN Induces Early Transcriptional and Metabolic Changes in Salt-Stressed *A. thaliana* Plants

**FIGURE 4:**
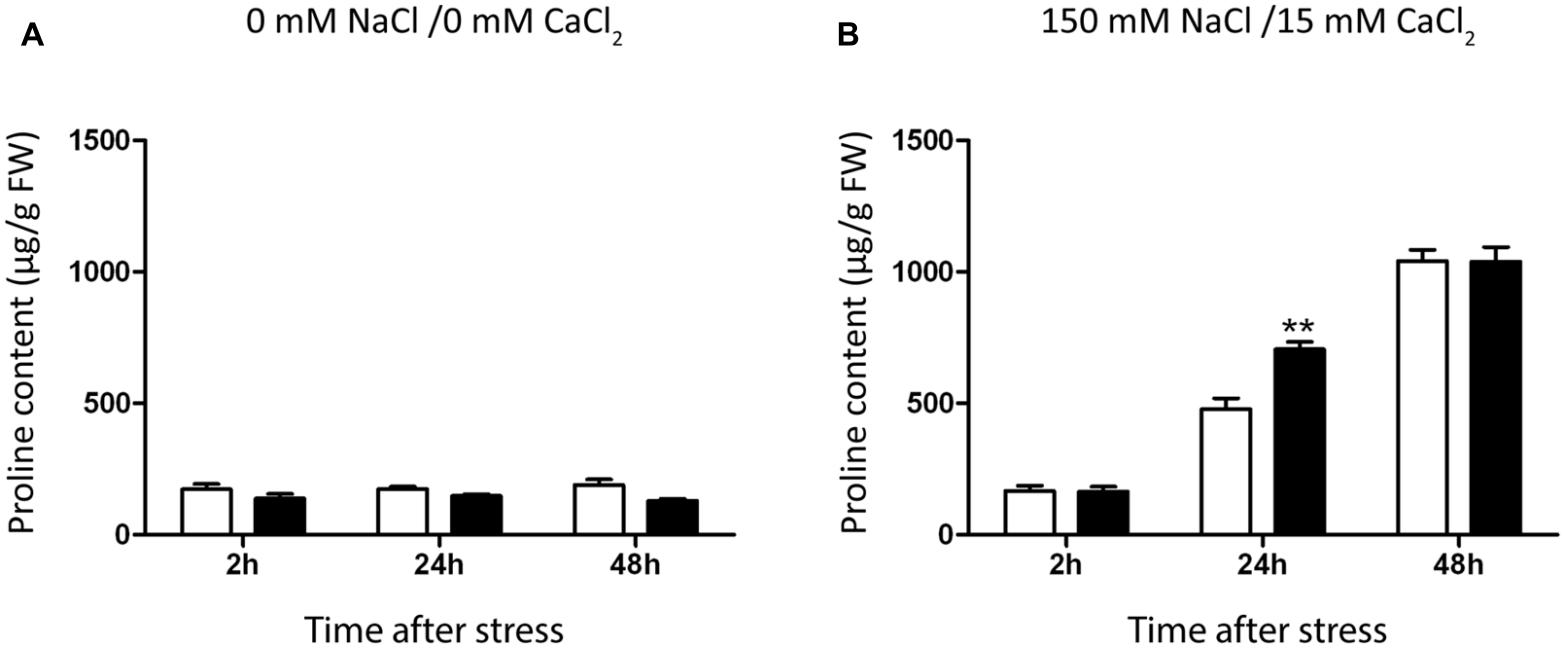
**Effect of *B. phytofirmans* PsJN on *A. thaliana* proline accumulation.** Graphic representation of proline levels in *A. thaliana* plants treated with or without *B. phytofirmans* PsJN, and transplanted at 11 DAS to half strength MS media with **(B)** or without **(A)** addition of 150 mM NaCl/15 mM CaCl_2_. Proline was extracted 2, 24, and 48 h after transplantation. Data are means ± 1 SE of at least three biological replicates. Asterisks indicate significant differences amongst treatments (Two-way ANOVA, *p* < 0.05; Bonferroni test, ^∗∗^*P* < 0.05).

**FIGURE 5:**
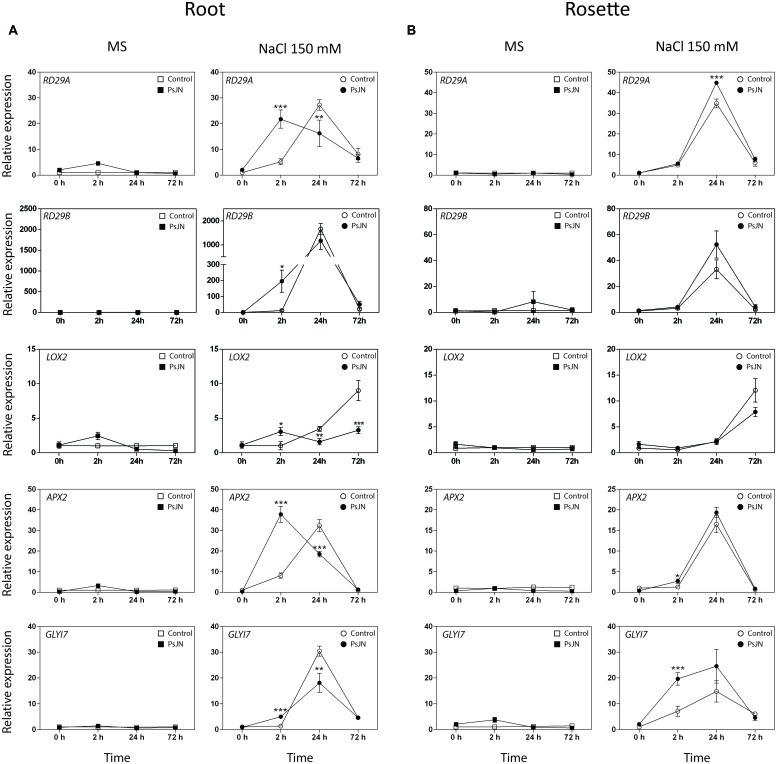
**Effects of *B. phytofirmans* on *A. thaliana* abiotic stress responsive genes.** Quantitative RT-PCR determinations of relative expression levels of the genes: *RD29A* (*Responsive to Dessication 29A*); *RD29B* (*Responsive to Dessication 29B*); *LOX2* (*Lipoxigenase 2*); *APX2* (*Ascorbate Peroxidase 2*); *GLYI7* (*Glyoxalase I 7*), in roots **(A)** and rosettes **(B)** of *A. thaliana* plants treated with or without strain PsJN, and transplanted at 11 DAS to half strength MS media with or without addition of 150 mM NaCl/15 mM CaCl_2_. RNA was extracted before transplantation (0 h) and after 2, 24, and 72 h in transplant media. Data are means ± 1 SE of at least three biological replicates. Asterisks indicate significant differences amongst treatments (Two-way ANOVA, *p* < 0.05; Bonferroni test, ^∗^*P* < 0.1; ^∗∗^*P* < 0.05; ^∗∗∗^*P* < 0.01).

To study the molecular and metabolic mechanisms behind the enhancement of salt-stress tolerance in PsJN inoculated plants, the content of the osmoprotectant molecule proline within plant tissues (Sneha et al., 2013) was measured 2, 24, and 48 h after transplant to MS or saline medium in both inoculated and non-inoculated plants (**Figure 4**). Also, the temporal expression patterns of genes related to general abiotic stresses were evaluated in roots and rosettes of plants in the early phase of short-term salt-stress treatments (**Figure 5**).

Proline content was not altered in plants transferred to MS1/2 media (**Figure 3A**), but augmented significantly 24 h after stress treatment, reaching a maximum value after 48 h (**Figure 4B**). Inoculated plants accumulated 47% more proline than control plants after 24 h of salt-stress (**Figure 4B**). At 48 h proline contents were comparable in both inoculated and control plants (**Figure 4B**).

Quantitative RT-PCR was used for transcriptional analysis of the genes: *Relative to Dessication A* (*RD29A*) and *B* (*RD29B*); *Lipoxygenase 2* (*LOX2*); *Plant-defensin 1.2* (*PDF1.2*); *Ascorbate Peroxidase 2* (*APX2*) and *Glyoxalase I 7* (*GLYI7*), (Yamaguchi-Shinozaki and Shinozaki, 1994; Thornalley, 1996; Davletova et al., 2005; Abogadallah, 2010; Leon-Reyes et al., 2010; Mustafiz et al., 2011; **Figure 5**). Expression of *PDF1.2* was not detected in roots. In rosettes, the transcript level of the gene was slightly affected by inoculation, and significantly up-regulated in the stressed and inoculated plants 24 h post-stress (Supplementary **Figure S2**). The transcription levels of the five other genes were not altered in roots or rosettes when plants were transferred to MS1/2 media (**Figures 5A,B**). Interestingly, in roots under salt-stress, inoculated plants presented an accelerated up-regulation of the *RD29A, RD29B, APX2*, and *GLYI7* at 2 h post-stress (up to 196 times in the case of *RD29B*, **Figure 5A**). In rosettes, plants treated with strain PsJN showed a clear up-regulation for *GLYI7*, and a minor but significant effect for *APX2* at 2 h post-treatment, and at 24 h for *RD29A* (**Figure 5B**). In the case of *LOX2*, the inoculation produced and up-regulation after 2 hours of stress, but remarkably after 24 and specially 72 h post-stress, a down-regulation of this gene was observed in roots (**Figure 5A**).

### *Burkholderia phytofirmans* PsJN is Associated with Early Changes in Transcriptional Response of Ion Transporter Genes in *A. thaliana* Plants under Salt-Stress

**FIGURE 6:**
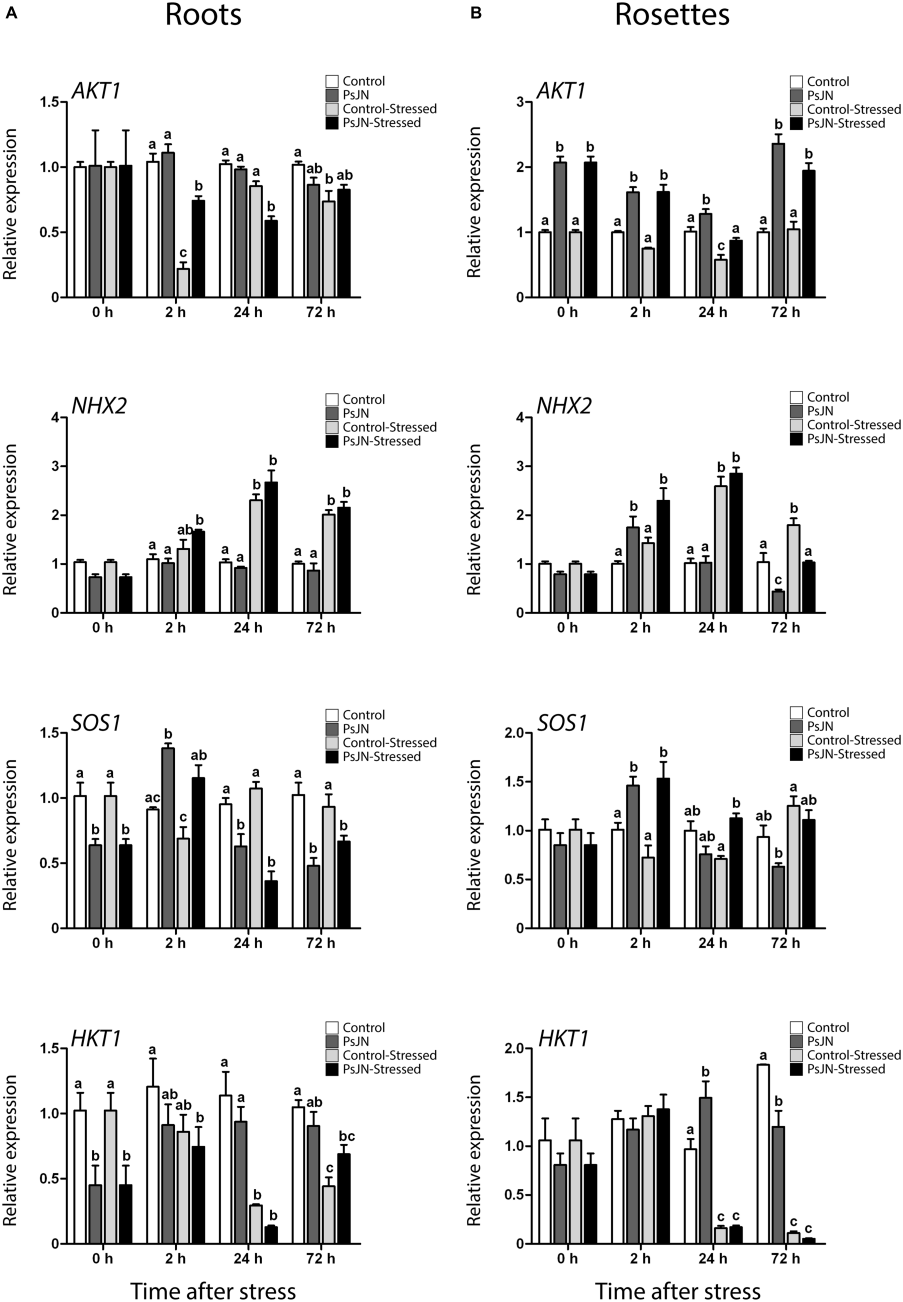
**Effect of *B. phytofirmans* PsJN on *A. thaliana* ion transporters transcription after short-term exposure to salt stress.** Quantitative RT-PCR determinations of relative expression levels of the genes: *AKT1 (Arabidopsis K^+^ Transporter 1*); *NHX2* (*Sodium Hydrogen Exchanger 2*); *SOS1* (*Salt Overly Sensitive 1*); and *HKT1* (*High-Affinity* K^+^ *Transporter 1*), in roots **(A)** and rosette **(B)** of *A. thaliana* plants treated with or without strain PsJN, and transplanted at 11 DAS to half strength MS media with or without addition of 150 mM NaCl/15 mM CaCl_2_. RNA was extracted before transplantation (0 h) and after 2, 24, and 72 h in transplant media. Data are means ± 1 SE of at least three biological replicates. Different letters represent significant differences between treatments at each time point (Two-way ANOVA, *p* < 0.05; Bonferroni test, *P* < 0.05).

To test for transcriptional differences in ion transporter genes in inoculated plants under salt-stress, the transcript level of *Arabidopsis K^+^ Transporter 1* (*AKT1*), *Sodium Hydrogen Exchanger 2* (*NHX2), Arabidopsis Salt Overly Sensitive 1* (*SOS1*), and *High-Affinity K^+^ Transporter 1* (*HKT1*) were measured (**Figure 6**). Plants were treated as described in the “Materials and Methods” section for RNA extraction and transcript level determination.

*Arabidopsis K^+^ Transporter 1* is a potassium plasma membrane transporter involved in root K^+^ uptake at any extracellular concentration above 10 μM (Sentenac et al., 1992; Lagarde et al., 1996). Here, *AKT1* transcript level depended on the interaction between the effects of salinity and bacteria in roots (**Figure 6A**). After 2 h of stress, salinity down-regulated this gene; an effect that was stronger in non-inoculated plants. After 24 h of stress in roots, treatment with PsJN down-regulated *AKT1* (**Figure 6A**). In rosettes, *AKT1* was significantly up-regulated by PsJN treatment in *A. thaliana* at all time points, while salinity seemed to down-regulate the expression of this gene after 24 h of stress (**Figure 6B**).

*Sodium Hydrogen Exchanger 2* is a vacuolar Na^+^, K^+^/H^+^ antiporter involved in ion compartmentalization in normal and salt-stress conditions (Jiang et al., 2010; Leidi et al., 2010). In roots, *NHX2* transcription depended only on the effect of salt, which up-regulated this gene at 2, 24, and 72 h post stress (**Figure 6A**). Transcript level on rosettes showed three different behaviors after salt-stress. Firstly, after 2 h of salt-stress, PsJN treatment up-regulated *NHX2* in a salt-independent manner (**Figure 6B**). Then, after 24 h of stress, salinity up-regulated this gene independently of inoculation (**Figure 6B**). Finally, at 72 h the expression depended on the interaction of salinity and PsJN, where bacteria down-regulated the gene (**Figure 6B**).

*Arabidopsis Salt Overly Sensitive 1* is a plasma membrane Na^+^/H^+^ antiporter (Zhu et al., 1998; Shi et al., 2002). It participates in sodium expulsion from the cytoplasm in salt-stress context (Zhu et al., 1998; Shi et al., 2002). Inoculation down-regulated *SOS1* in roots before exposure to stress (**Figure 6A**). In this tissue, transcript accumulation after transplant depended only on the effect of bacteria, which up-regulated this gene after 2 h, and then repressed it after 24 and 72 h (**Figure 6A**). In rosettes, plants treated with strain PsJN showed a significant increase in *SOS1* transcript 2 and 24 h after the transplant to saline media (**Figure 6B**).

*High-Affinity K^+^ Transporter 1* is a plasma membrane sodium transporter (Maser et al., 2002; Davenport et al., 2007). It has been related to sodium unloading from xylem at root level, preventing its movement to leaves under salt-stress conditions (Maser et al., 2002; Davenport et al., 2007). Under these experimental conditions the gene was down-regulated in roots by bacteria treatment before exposure to stress, then the inoculation down-regulated it after 2 h in saline media, while the same was observed at 24 h in non-inoculated plants (**Figure 6A**). In rosettes, the gene was slightly up-regulated in non-stressed and inoculated plants at 24 h, while it was markedly down-regulated by the salt-stress at 24 and 72 h independently of the inoculation (**Figure 6B**).

### *Burkholderia phytofirmans* Modifies *A. thaliana* Expression of Ion Transporters and Detoxification Genes after Long-Term Exposure to Salt-Stress

**FIGURE 7:**
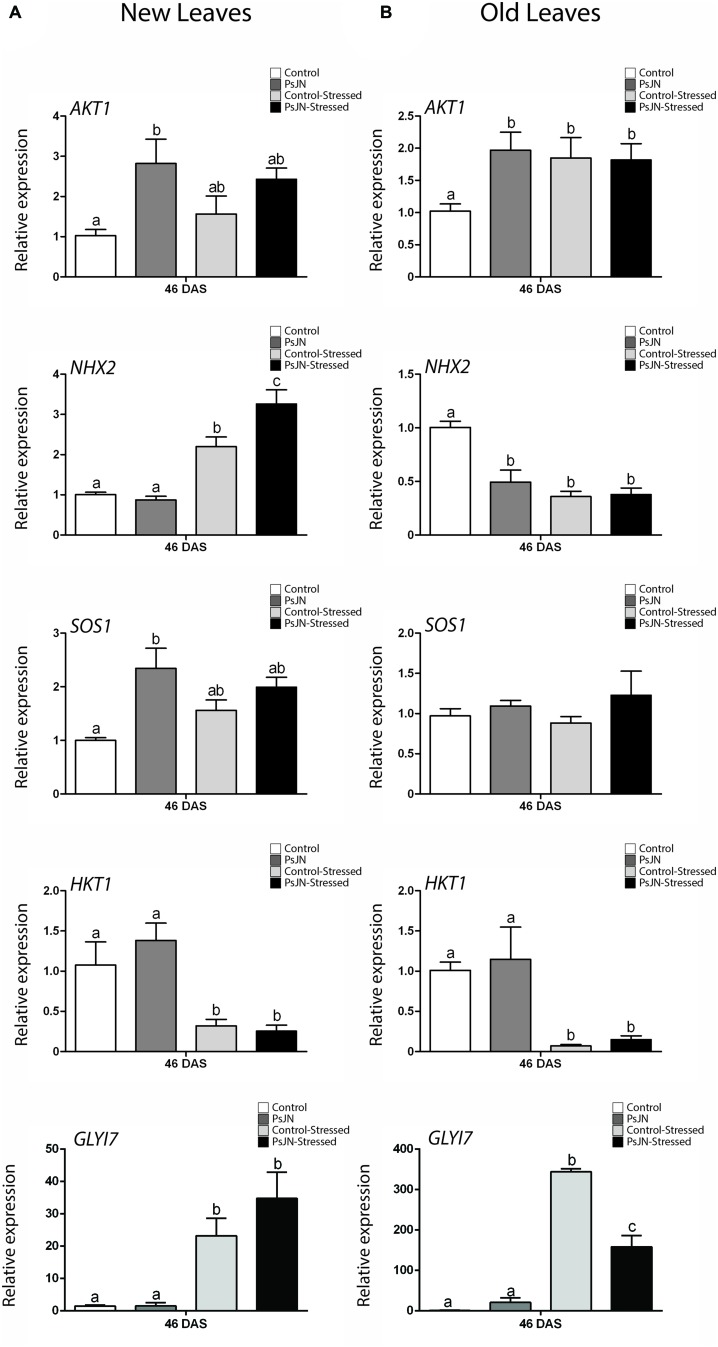
**Effect of *B. phytofirmans* PsJN on *A. thaliana* salt-stress related genes transcription after long-term exposure to salt stress.** Quantitative RT-PCR determinations of relative expression levels of the genes: *AKT1*; *NHX2*; *SOS1*; *HKT1*, and *GLYI7*, in new **(A)** and old leaves **(B)** of *A. thaliana* plants treated with or without strain PsJN, and transplanted at 11 DAS to soil. After 7 days of acclimation plant were irrigated with water with or without addition of 150 mM NaCl/15 mM CaCl_2_. RNA was extracted 35 days after transplantation (46 DAS). Data are means ± 1 SE of at least three biological replicates. Different letters indicate significant differences between treatments (Two-way ANOVA, *p* < 0.05; Bonferroni test, *P* < 0.05).

To determine if treatment with *B. phytofirmans* PsJN had long-term effects on the transcription of the ion transporters genes *AKT1*, *NHX2*, *SOS1*, and *HKT1*, plants were treated as described in the “Materials and Methods” section. For RNA extraction, the oldest and the newest leaves of each plant were selected, and RNA extractions were performed at a point were salt-stress had not stopped plant growth but stress signs were noted (46 DAS, **Figure 3**). As noticed before, *AKT1* transcript accumulation was up-regulated by PsJN treatment both in new and old leaves at 35 days after stress (**Figures 7A,B**). The gene was up-regulated also by salt-stress only in old leaves (**Figure 7B**). The vacuolar transporter *NHX2*, showed an up-regulation due to salinity in new leaves. Interestingly, a stronger and significant up-regulation was observed when plants were inoculated with strain PsJN (**Figure 7A**). In old leaves this gene was similarly down-regulated by bacteria and salt-stress (**Figure 7B**). *SOS1* expression was not altered in old leaves and PsJN up-regulated this gene in new leaves in non-stressed plants (**Figure 7A**). *HKT1* transcript level, consistent with what was observed before, only depended on the effect of salinity that significantly down-regulated this gene, both in new and old leaves (**Figures 7A,B**). Finally, the expression of some of the early-stress responsive genes was analyzed after long-term exposure to stress. Expression of *RD29B, PDF1.2*, and *LOX2* was not affected by salinity or PsJN inoculation at this time in new leaves (Supplementary **Figure S3**) and was not detected in old leaves (data not-shown). The detoxification gene, *GLYI7*, was up-regulated by salinity and not by PsJN in new leaves (**Figure 7A**). Notably, in old leaves, salinity also produced an up-regulation of the gene, but this effect was significantly lower when plants were treated with strain PsJN (**Figure 7B**).

## Discussion

It is well known that some PGPRs can enhance the tolerance of certain plant species to salt-stress (Chang et al., 2014). Most studies are mainly focused on physiological or metabolic parameters, basing their results on changes in growth, nutrient uptake, and/or synthesis of stress related compounds (Ait Barka et al., 2006; Upadhyay et al., 2011). In terms of transcriptional changes, there is literature that relates the effects of PGPRs with a rapid activation of the immune system, leading to a primed or faster and stronger response to biotic (Van Loon et al., 1998; Pieterse et al., 2013) and abiotic stress (Induced Systemic Tolerance, IST, Yang et al., 2009; Theocharis et al., 2012; Kim et al., 2014). Unfortunately, there is little evidence about the links between PGPR inoculation and ion transporter genes related to salt tolerance in plants (Zhang et al., 2008). Also, transcriptional analysis in the vast majority of salt-stress studies is focused on the first hours after stress, but little is known about the molecular effects of salinity after a long-term exposure (Geng et al., 2013; Kim et al., 2014). In this study, we showed that *B. phytofirmans* PsJN enhances *A. thaliana* growth and salt tolerance in various experimental schemes during the whole life cycle of the plant. Interestingly, this effect seems to be related with an acceleration of the natural metabolic and transcriptional response to salt-stress in *Arabidopsis* and also with the long-term regulation of some genes. Moreover, the transcription of some important ion transporters related to salt-stress tolerance was modified in plants treated with strain PsJN and, remarkably, these changes were observed both in plants exposed or not to salt-stress.

### Effects of *B. phytofirmans* on *A. thaliana* Growth after Short and Long-Term Salt-Stress

*Burkholderia phytofirmans* PsJN is a PGPR capable of producing positive effects on *Arabidopsis* (Poupin et al., 2013; Zuniga et al., 2013) and in horticultural crops such as potato and grape (Compant et al., 2005; Sessitsch et al., 2005; Da et al., 2012; Mitter et al., 2013). Also, it has been related with biotic and abiotic induced tolerance in plants like grape, maize, and wheat (Ait Barka et al., 2006; Bordiec et al., 2011; Fernandez et al., 2012; Naveed et al., 2014). Here, treatment with *B. phytofirmans* PsJN promoted the growth of *A. thaliana* in saline media at high salt concentrations, from 150 mM NaCl/15 mM CaCl_2_ to 250 mM NaCl/25 mM CaCl_2_. This effect was only noted when bacteria were metabolically active in agreement with previous work of Poupin et al. (2013), that reported that inactivated PsJN cannot induce growth in *A. thaliana*. This suggests that growth promotion and/or salt-stress tolerance induction is not related to mere plant recognition of bacterial molecular determinants, either expressed in the bacterial surface or released during heat inactivation, and that metabolically active bacteria are required to induce those changes. Regarding the molecular determinants of PsJN that trigger induced salt-tress tolerance in plants, we have recently found evidence of a possible role of volatile organic compounds (Ledger et al., unpublished). When plants were inoculated with strain PsJN the rate of recovery, after 7 days of stress, was higher than in the non-inoculated plants. Interestingly, the development of inoculated and stressed plants was comparable to the control group (non-stressed). Remarkably, an early inoculation at germination induced salt-stress tolerance when the stress was maintained over time, suggesting the effects of PsJN trigger long-term changes in plants.

### Transcriptional and Metabolic Effects of *B. phytofirmans* in *A. thaliana* Early Response to Salt-Stress

*Relative to Dessication A* (*RD29A*) and *RD29B* are commonly used as a stress responsive reporter genes, *RD29A* promoter region has one ABA responsive *cis*-acting element (ABRE) and many ABA-independent dehydration responsive elements (DRE/CRT), responding mainly to the ABA-independent pathway. On the other hand, the *RD29B* promoter only has ABRE elements, being controlled mainly by ABA (Yamaguchi-Shinozaki and Shinozaki, 1994; Hu et al., 2013). ABRE binding transcription factors (AREB) are a superfamily that responds mainly to drought and high salinity stresses (Li et al., 2014). While DRE binding (DREB) transcription factors respond to cold and osmotic stresses (Li et al., 2014). Both AREB and DREB elements promote the transcription of many genes related to stress response (Finkelstein et al., 2002; Akhtar et al., 2012). It has also been reported that *RD29A* and *RD29*B encodes for similar proteins, but the first respond mainly to cold and drought while the second one is highly induced by salinity (Msanne et al., 2011). *B. phytofirmans* PsJN treatment accelerated the transcript accumulation of *RD29A* and more dramatically of *RD29B* in roots. These results can be related to a faster sensing of osmotic and/or salt-stress via ABA or dehydration signaling. Interestingly, the highest effect of bacterial treatment on saline media in *RD29B* (around 196 times) could be associated with an active ABA pathway in roots induced by PsJN. In rosette, *RD29A* expression was up-regulated by the bacterial treatment, while no significant effect was noted on *RD29B* expression, this could indicate the activation of an ABA-independent pathway induced by PsJN in the aerial zone. Either way, the acceleration on expression of both genes probably leads to a faster response of ABRE and/or DREB elements, and consequently, a primed transcription of stress responsive genes (**Figure 5**).

*Ascorbate Peroxidase 2* enzyme that reduces the reactive oxygen species H_2_O_2_ to H_2_O by the oxidation of ascorbate (Abogadallah, 2010). It has been documented that ROS are both helpful and harmful to plants in a stress situation, because they act as second messenger in stress response, but also their accumulation drives to oxidation of several molecules and cell damage (Apel and Hirt, 2004; Golldack et al., 2014). When ROS accumulate at later stages, or are formed as a consequence of mitochondrial damage, jasmonic acid (JA) synthesis is over induced together with an autocatalytic oxidative burst (Ismail et al., 2014). Therefore, plants require a tight regulation between formation and scavenging of ROS, in order to perceive the stress signal, but also to be capable of resisting it (Apel and Hirt, 2004; Bhattacharjee, 2012; Garcia de la Garma et al., 2015). Bharti et al. (2013) observed that foliar Ascorbate Peroxidase activity was increased by PGPR treatment, and this was associated with general salt tolerance augmentation on Bacopa (*Bacopa monnieri*). Here, the inoculation of *A. thaliana* plants with the strain PsJN accelerated the transcript accumulation of *APX2* in roots and up-regulated it in the rosettes of salt-stressed plants (**Figure 5**). Interestingly, *LOX2* that codes for a Lipoxygenase involved in JA biosynthesis, presented an up-regulation after 2 h of stress, but after 24 and specially 72 h post-stress, a down-regulation of this gene was observed. Similarly, *PDF1.2* (that encodes an ethylene and jasmonate-responsive plant defensin) presented a temporal up-regulation in stressed rosettes (24 h) and then returned to a similar level than the non-inoculated plants. This is in accordance with our previous results showing that *LOX2* and *PDF1.2* are two of the 408 genes with altered transcription in the transcriptome of PsJN inoculated plants (Poupin et al., 2013). These expression patterns on the JA and/or ethylene-related genes reflect the complexity of the hormonal-related responses, where genes can be controlled through distinct pathways or with a different timing, being the crosstalk between hormones one of the crucial aspects in the final outcome of stress response. Thus, PsJN treatment could be incrementing APX2 activity on roots and shoots, improving plant capacity for ROS scavenging, while reducing the expression of the JA biosynthesis-related gene *LOX2*. This could lead, according to the literature (Apel and Hirt, 2004; Abogadallah, 2010), to a better sensing of ROS as a second messenger deriving to a more effective response to salt-stress. Future quantifications of APX2 activity, JA levels and ROS quantities could support this hypothesis.

*Glyoxalase I 7* is a highly abiotic-stress inducible isoform of Glyoxalase I, an enzyme part of the glyoxalase pathway (Thornalley, 1996; Mustafiz et al., 2011). The main function of this pathway is to detoxify the cells of methylglyoxal, a toxic compound that is accumulated during stressful conditions (Thornalley, 1996). In tobacco and tomato, lines overexpressing *GLYI* and *GLYII*, showed an increment in salt tolerance (Singla-Pareek et al., 2003; Álvarez Viveros et al., 2013). Similarly in *A. thaliana*, mutation in *GLYII* gene inhibits growth under saline conditions, while overexpressing lines of the same gene showed a modest protective effect (Devanathan et al., 2014). Here, *GLYI7* transcription was anticipated by PsJN treatment in roots and up-regulated in shoots of salt-stressed plants.

One of the main mechanisms to alleviate osmotic-stress is the synthesis of osmoprotectant molecules, proline being one of the most important (Yoshiba et al., 1995). It has been documented that proline accumulation is highly inducible by salinity, especially in the first 24 h after salt exposure (Verslues and Sharma, 2010). Ait Barka et al. (2006) demonstrated that *B. phytofirmans* treatment confers chilling tolerance to *Vitis vinifera* plants, and also produced an augmentation in the accumulation of proline in these plants. In this investigation, the proline accumulation was studied in a temporal context and strain PsJN accelerated its accumulation during the first 24 h after salt-stress treatment.

It has been recently proposed that the relation between adaptive salt signaling and destructive salt accumulation depends on the timing of the events triggered by the salt-stress. An efficient response could constrain a JA signaling through the activation of ABA (Ismail et al., 2014). Here, an anticipation of proline content augmentation, together with an anticipation of *RD29A, RD29B, APX2*, and *GLYI7* transcription, was observed during the first 24 h under salt-stress in the roots of inoculated plants. Remarkably, the gene showing the highest up-regulations was *RD29B*, which is mainly regulated by ABA-dependent pathways. Similar results, but in some genes with slower kinetics, were obtained in rosettes. Also, the expression of *LOX2*, related with JA biosynthesis was mainly down-regulated in the inoculated and stressed plants. Interestingly, these genes were not induced when plants were in control conditions. Therefore, plants inoculated at germination responded faster to a later salt-stress, this could be indicating an induction of a priming in inoculated plants by *B. phytofirmans* PsJN that lead to a better response to this abiotic stress. Notably, the regulation by PsJN in the expression of some of the genes involved in stress-alleviation such as *GLYI7*, at least in old leaves, was maintained over time.

### Effects of *B. phytofirmans* in *A. thaliana* Ion Transporters Transcription under Short and Long-Term Salt-Stress

Many studies have focused on revealing the mechanisms behind salt tolerance in plants (Zhang and Shi, 2013; Gupta and Huang, 2014). Two of the most important determinants for salt tolerance discovered so far are: the maintenance of a high K^+^/Na^+^ ratio, and the conservation of a low salinity concentration in the cytoplasm (Zhang and Shi, 2013; Gupta and Huang, 2014). In normal conditions potassium is the most abundant intracellular cation (Britto and Kronzucker, 2008). This ion is used as co-factor for many enzymatic reactions and it is also important for controlling the stomatal aperture (MacRobbie, 1998; Maathuis, 2009). Potassium uptake is mediated by ion transporters like *AKT1*, which mediates the uptake of this ion at any extracellular concentration above 10 μM (Nieves-Cordones et al., 2014). Once inside the cell, K^+^ is stored in the vacuole by the action of antiporters as *NHX2* (Jiang et al., 2010; Leidi et al., 2010). When plants are exposed to salt-stress, there is an increase of sodium uptake that reduces the K^+^/Na^+^ ratio, leading to toxicity (Hasegawa, 2013). When sodium enters the roots it moves toward the xylem and up to the leaves, which are extremely sensitive to Na^+^ toxicity. The sodium accumulation in leaves, at toxic levels, produces enzymatic malfunction, generation of ROS, and finally cell death (Hasegawa, 2013). In order to tolerate the effects of sodium toxicity, most plants have various ion transporters that maintain the ionic homeostasis inside the cell (Zhang and Shi, 2013; Gupta and Huang, 2014). First, there are transporters belonging to the NHX family (Bassil and Blumwald, 2014), that are believed to reduce Na^+^ concentration, driving this ion inside the vacuole (NHX2) or expelling it to the apoplast (SOS1), (Bassil and Blumwald, 2014; Fan et al., 2014). *A. thaliana* mutants in *SOS1* accumulate more sodium in leaves and roots, and are more sensitive to salinity (Shi et al., 2002). NHX2 is a vacuolar antiporter that drives Na^+^ or K^+^ into the vacuole and evicts H^+^ (Jiang et al., 2010; Leidi et al., 2010). *NHX2* is one of the most abundantly transcribed genes in *A. thaliana*, and its expression is regulated by salt (NaCl, LiCl, KCl), osmotic-stress and ABA (Shi et al., 2002; Aharon et al., 2003). NHX2 is only absent in meristematic cells of the root tip (Shi et al., 2002). It is known that overexpression of *NHX1* increases salt tolerance in *A. thaliana* plants, and that mutants in *nhx1* and *nhx2* are more sensitive to salinity (Barragan et al., 2012; Fan et al., 2014). The mechanism by which this transporter improves salt tolerance is not clear; some researchers attribute the effect to a better K^+^ retention, while others think that this transporter improves the sodium compartmentalization (Barragan et al., 2012; Fan et al., 2014).

Other transporters are responsible for the selective accumulation of Na^+^. For example HKT1, which is specifically localized in the root XPC, unloads Na^+^ from the vascular conducts to prevent its accumulation on leaves (Sunarpi et al., 2005). It is not completely clear how *HKT1* is regulated, but some reports correlated the presence of ROS to a reduction of Na^+^ in leaves, supposedly via HKT1 activity (Jiang et al., 2012). There is also evidence to support a repression of *HKT1* by ABA, via the transcriptional factor ABI4 that binds to the *HKT1* promoter (Shkolnik-Inbar et al., 2013). Experiments with *HKT1* cell-specific overexpressing mutants, showed that the activity of this transporter is crucial for salt tolerance, but only when it is overexpressed in the root stele (Moller et al., 2009).

Among the general transcriptional effects of PsJN in inoculated plants (Poupin et al., 2013) in this study, it was found that the bacterium also regulate the expression of important ion-homeostasis related genes after short and long-term exposure to salt-stress. Some of these genes were mainly regulated by the bacterial inoculation, while the others by the interaction between the inoculation and the salt-stress exposure. The expression patterns were differently affected in roots and shoots. The transcription of *AKT1* on rosettes, and *SOS1* and *HKT1* on roots was modified by PsJN treatment before salt treatment. This means that PsJN affects some genes related to salt-stress in a manner independent of the stress challenge. After stress, genes presented three different patterns. First, there were genes that showed an acceleration of their normal response (*NHX2* and *SOS1* in rosettes, and *SOS1* and *HKT1* in roots). This is similar to what was observed in general stress responsive genes, where natural transcriptional response was developed early, suggesting a faster or primed response to stress in inoculated plants. In the second group, there were genes that were not affected by bacterial-inoculation (*HKT1* in rosette and *NHX2* in roots). Finally, *AKT1* showed a different behavior to the one mentioned above. In roots, PsJN-inoculation appeared to delay the response of this gene. While in shoots, *AKT1* was up-regulated at all the time points, including before salt-stress. Down-regulation of *SOS1* and *HKT1* before stress could be related to a general reduction in Na^+^ levels, making xylem Na^+^ unloading and expulsion a less necessary task.

After long-term exposure to salinity, *AKT1, NHX2*, and *SOS1* were significantly affected by *B. phytofirmans* treatment. In old leaves, inoculated non-stressed plants presented similar patterns to the stressed plants regarding *AKT1* and *NHX2* genes. This could mean that bacterial and salt treatments produce a similar transcriptional effect, but in the case of PsJN, it is not linked to damage, so plants could be responding to a non-harmful signal in a way that can also protect them in the eventuality of being affected by salt-stress. In new leaves *AKT1* and *SOS1* expression was up-regulated by PsJN treatment when plants where not stressed, while salinity did not significantly affect the expression of these genes. On the other hand, *NHX2* expression was not affected in non-stressed plants, but was significantly up-regulated by salinity. Interestingly, this effect was potentiated by PsJN inoculation.

As has been discussed, the response timing of plants under stress is crucial to trigger specific pathways that lead to an effective adaptation. Under these experimental conditions, an early inoculation with strain PsJN increased salt-stress tolerance in plants under short and long-terms of stress exposure. The inoculation accelerated salt-stress molecular responses of *A. thaliana* involved in ABA-dependent pathways; ROS scavenging and detoxifying; down-regulated a gene related with JA biosynthesis and modified the expression of genes specifically related with ion homeostasis. A differential transcriptional effect was observed in roots and shoots. Especially in the early-stress responsive genes, the roots seem to present an early and faster response than the aerial zone. Which is consistent with the onset of stress with salt accumulation beginning at the root level, and reaching aerial tissues at a later stage? Overall, the stronger and faster molecular changes induced by the inoculation with *B. phytofirmans* PsJN suggest a priming effect of this strain in the inoculated plants. In some genes the regulation in their expression was maintained over time. This may lead to salt-stress tolerance and could explain the induction of long-term tolerance to this abiotic stress. These findings contribute to a better understanding of the molecular mechanisms underlying salt tolerance enhancement induced by beneficial bacteria, reflecting a complex array of hormonal crosstalk and molecular plant responses that leads to the outcome of the stress situation in inoculated plants. Also, they may open up new alternatives for a strategy against salinity limitations on crop culture, as well as new approaches to discover mechanisms involved in stress tolerance in plants.

## Conflict of Interest Statement

The authors declare that the research was conducted in the absence of any commercial or financial relationships that could be construed as a potential conflict of interest.

## Supplementary material

Figure S1 **Effects of killed *Burkholderia phytofirmans* PsJN on *Arabidopsis thaliana* growth *in vitro.*** Graphic representation of relative fresh weight **(A)**, rosette area **(B)** and primary root length **(C)** of *A. thaliana plants* treated with *B. phytofirmans* PsJN, heat-inactivated strain PsJN (K-PsJN) or without inoculation (control), and transplanted at 11 days after sowing (DAS) to MS with or without addition of 150 mM NaCl/15 mM CaCl_2_. Data were collected 7 days after transplantation. Data are means ±1 SE of at least 20 plants per treatment (One way ANOVA, *p* < 0.05; Bonferroni test, **P* < 0.1, ****P* < 0.01).

Figure S2 **Effects of *Burkholderia phytofirmans on Arabidopsis thaliana PDF1.2* gene transcription.** Quantitative RT-PCR determinations of relative expression levels of *PDF1.2* (*Plant Defensin 1.2*) in rosettes of *A. thaliana* plants treated with or without strain PsJN, and transplanted at 11 days after sowing (DAS) to half strength Murashige Skoog media (MS) with or without addition of 150 mM NaCl/15 mM CaCl_2_. RNA was extracted before transplantation (0h) and after 2, 24 and 72 hours in transplant media. Data are means ±1 SE of at least 3 biological replicates. Asterisks indicate significant differences amongst treatments (Two way ANOVA, *p* < 0.05; Bonferroni test, **P* < 0.1).

Figure S3 **Effect of *Burkholderia phytofirmans* PsJN on *Arabidopsis thaliana* abiotic stress responsive genes after long-term exposure to salt stress.** Quantitative RT-PCR determinations of relative expression levels of the genes: RD29B (*Responsive to Dessication 29B*); *LOX2* (*Lipoxigenase 2*); *PDF1.2* (*Plant Defensin 1.2*), in new leaves of *A. thaliana* plants treated with or without strain PsJN, and transplanted at 11 days after sowing (DAS) to soil. After 7 days of acclimation plant were irrigated with water with or without addition of 150 mM NaCl/15 mM CaCl_2_. RNA was extracted 35 days after irrigation started (46 DAS). Data are means ±1 SE of at least 3 biological replicates (Significant differences were not detected in any of the treatments in the analyzed genes, ANOVA, *P* < 0.05).
