# Supplementary material for: Burkholderia phytofirmans PsJN induces long-term metabolic and transcriptional changes involved in Arabidopsis thaliana salt tolerance
Source: Front Plant Sci. 2015 Jun 23;6:466. doi: 10.3389/fpls.2015.00466 (PMC4477060; doi:10.3389/fpls.2015.00466)
Supplement: Supplementary file 1 [file Table_1.DOCX]

| Table S1. List of primers. | | | | | |
| --- | --- | --- | --- | --- | --- |
| **Gene** | **Locus** | **Forward** | **Reverse** | **Tm** | **Reference** |
| AKT1 | At2g26650 | GCTTGCACGGGGTAAAATGG | GTCTGCGTGGTACTCCAACA | 57.0 | Designed here |
| APX2 | At3g09640 | TGGTCGGATGGGACTCAAT | AAGAGCCTTGTCGGTTGGT | 58.5 | Xie *et al*., 2012 |
| GLYI7 | At1g80160 | CTACGATCGAGCCAGCGTTC | CCATGTCCAAACAACCACGC | 57.5 | Designed here |
| HKT1 | At4g10310 | CCTCTCACTTTGTATTGTATTG | AAGCATTAATATTATACTGGCC | 53.8 | Designed here |
| LOX2 | At3g45140 | ATCAACGCTCGTGCACGCCA | CCGCGGGTAAGCCTTCCTGG | 64 | Poupin et al., 2013 |
| LOX2 | At3g45140 | ATCAACGCTCGTGCACGCCA | CCGCGGGTAAGCCTTCCTGG | 64 | Poupin et al., 2013 |
| NHX2 | At3g05030 | GAATTCCTCAACGCAACTCAATCC | CTGCAGGGCGAACATTGTCATCTT | 54.2 | Aharon *et al*., 2003 |
| PDF1.2 | At5g44420 | CTTGTTCTCTTTGCTGCTTTCGAC | ATGCATTACTGTTTCCGCAAACC | 61 | Poupin et al., 2013 |
| RD29A | At5g52310 | ATCACTTGGCTCCACTGTTGTTC | ACAAAACACACATAAACATCCAAAGT | 57.0 | Bu *et al*., 2009 |
| RD29B | At5g52300 | GGAGTGAAGGAGACGCAACAAG | GGAATCCGAAAACCCCATAGTC | 58 | Designed here |
| SAND | At2g28390 | AACTCTATGCAGCATTTGATCCACT | TGATTGCATATCTTTATCGCCATC | 59 | Czechowski et al., 2005 |
| SOS1 | At2g01980 | CACTTCTGGGAAATGGTTGCA | TGCCTTCAGCAATGACAACAC | 56.0 | Bose et al., 2013 |

**Aharon GS, Apse MP, Duan SL, Hua XJ, Blumwald E. 2003.** Characterization of a family of vacuolar Na+/H+ antiporters in Arabidopsis thaliana. *Plant and Soil* **253**: 245–256.

**Bose J, Xie Y, Shen W, Shabala S. 2013.** Haem oxygenase modifies salinity tolerance in Arabidopsis by controlling K+ retention via regulation of the plasma membrane H+-ATPase and by altering SOS1 transcript levels in roots. *J Exp Bot* **64**(2): 471-481.

**Bu Q, Li H, Zhao Q, Jiang H, Zhai Q, Zhang J, Wu X, Sun J, Xie Q, Wang D, et al. 2009.** The Arabidopsis RING Finger E3 Ligase RHA2a Is a Novel Positive Regulator of Abscisic Acid Signaling during Seed Germination and Early Seedling Development. *Plant Physiol* **150**(1): 463-481.

**Czechowski, T., Stitt, M., Altmann, T., Udvardi, M.K., and Scheible, W.R.** (2005). Genome-wide identification and testing of superior reference genes for transcript normalization in Arabidopsis. *Plant Physiol* 139, 5-17. doi: 10.1104/pp.105.063743.

**Poupin, M.J., Timmermann, T., Vega, A., Zuniga, A., and Gonzalez, B.** (2013). Effects of the plant growth-promoting bacterium Burkholderia phytofirmans PsJN throughout the life cycle of Arabidopsis thaliana. *PLoS One* 8, e69435. doi: 10.1371/journal.pone.0069435.

**Xie Y, Mao Y, Lai D, Zhang W, Shen W. 2012.** H_2_ Enhances Arabidopsis Salt Tolerance by Manipulating ZAT10/12-Mediated Antioxidant Defence and Controlling Sodium Exclusion. *PLoS One* **7**(11): e49800.
