# Supplementary figures and images for: Burkholderia phytofirmans PsJN induces long-term metabolic and transcriptional changes involved in Arabidopsis thaliana salt tolerance
Source: Front Plant Sci. 2015 Jun 23;6:466. doi: 10.3389/fpls.2015.00466 (PMC4477060; doi:10.3389/fpls.2015.00466)

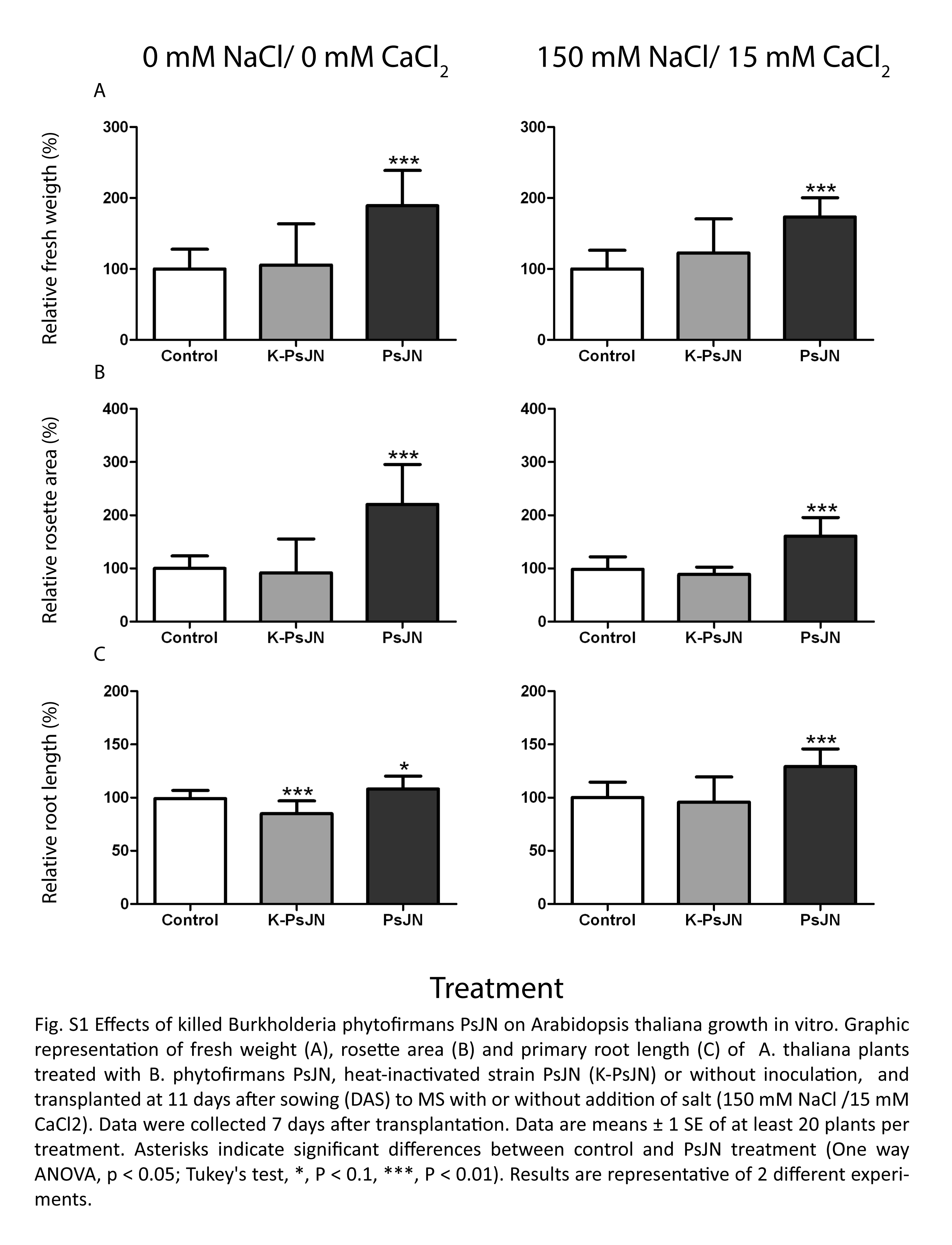

Supplement: Figure S1 — Effects of killed Burkholderia phytofirmans PsJN on Arabidopsis thaliana growth in vitro. Graphic representation of relative fresh weight (A), rosette area (B) and primary root length (C) of A. thaliana plants treated with B. phytofirmans PsJN, heat-inactivated strain PsJN (K-PsJN) or without inoculation (control), and transplanted at 11 days after sowing (DAS) to MS with or without addition of 150 mM NaCl/15 mM CaCl2. Data were collected 7 days after transplantation. Data are means ±1 SE of at least 20 plants per treatment (One way ANOVA, p < 0.05; Bonferroni test, *P < 0.1, ***P < 0.01). [file Image_1.TIF]

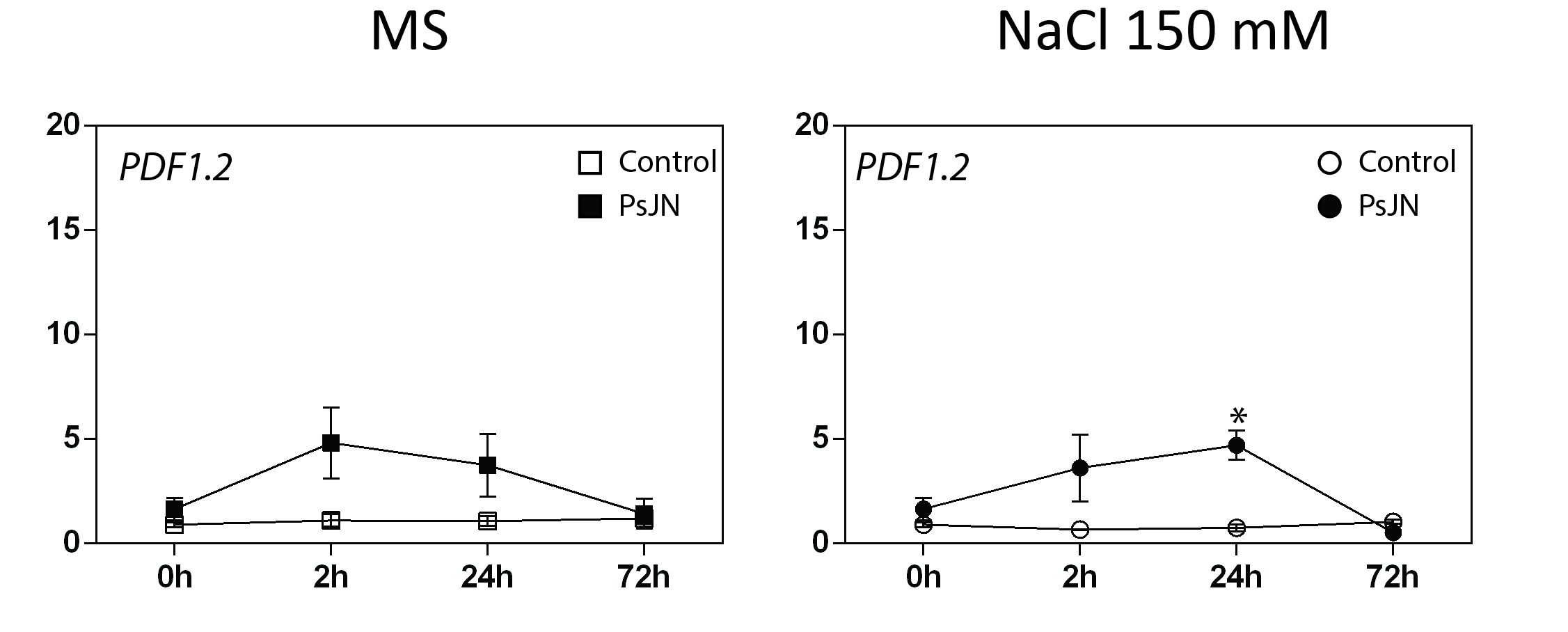

Supplement: Figure S2 — Effects of Burkholderia phytofirmans on Arabidopsis thaliana PDF1.2 gene transcription. Quantitative RT-PCR determinations of relative expression levels of PDF1.2 (Plant Defensin 1.2) in rosettes of A. thaliana plants treated with or without strain PsJN, and transplanted at 11 days after sowing (DAS) to half strength Murashige Skoog media (MS) with or without addition of 150 mM NaCl/15 mM CaCl2. RNA was extracted before transplantation (0h) and after 2, 24 and 72 hours in transplant media. Data are means ±1 SE of at least 3 biological replicates. Asterisks indicate significant differences amongst treatments (Two way ANOVA, p < 0.05; Bonferroni test, *P < 0.1). [file Image_2.TIFF]

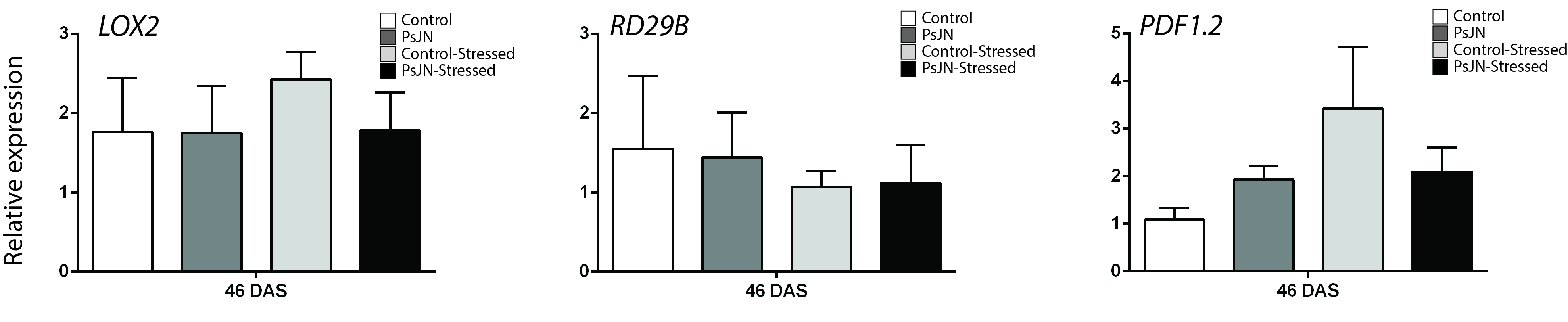

Supplement: Figure S3 — Effect of Burkholderia phytofirmans PsJN on Arabidopsis thaliana abiotic stress responsive genes after long-term exposure to salt stress. Quantitative RT-PCR determinations of relative expression levels of the genes: RD29B (Responsive to Dessication 29B); LOX2 (Lipoxigenase 2); PDF1.2 (Plant Defensin 1.2), in new leaves of A. thaliana plants treated with or without strain PsJN, and transplanted at 11 days after sowing (DAS) to soil. After 7 days of acclimation plant were irrigated with water with or without addition of 150 mM NaCl/15 mM CaCl2. RNA was extracted 35 days after irrigation started (46 DAS). Data are means ±1 SE of at least 3 biological replicates (Significant differences were not detected in any of the treatments in the analyzed genes, ANOVA, P < 0.05). [file Image_3.TIF]
